# Supporting Aboriginal and Torres Strait Islander Families to Stay Together from the Start (SAFeST Start): Urgent call to action to address crisis in infant removals

**DOI:** 10.1002/ajs4.200

**Published:** 2022-01-26

**Authors:** Catherine Chamberlain, Paul Gray, Debra Bennet, Alison Elliott, Marika Jackomos, Jacynta Krakouer, Rhonda Marriott, Birri O'Dea, Julie Andrews, Shawana Andrews, Caroline Atkinson, Judy Atkinson, Alex Bhathal, Gina Bundle, Shanamae Davies, Helen Herrman, Sue‐Anne Hunter, Glenda Jones‐Terare, Cathy Leane, Sarah Mares, Jennifer McConachy, Fiona Mensah, Catherine Mills, Janine Mohammed, Lumbini Hetti Mudiyanselage, Melissa O'Donnell, Elizabeth Orr, Naomi Priest, Yvette Roe, Kristen Smith, Catherine Waldby, Helen Milroy, Marcia Langton

**Affiliations:** ^1^ Centre for Health Equity The University of Melbourne Melbourne Vic. Australia; ^2^ Judith Lumley Centre La Trobe University Melbourne Vic. Australia; ^3^ The Lowitja Institute Carlton Vic Australia; ^4^ NGANGK YIRA: Murdoch University Research Centre for Aboriginal Health and Social Equity Murdoch WA Australia; ^5^ SNAICC ‐ National Voice for our Children Collingwood Vic. Australia; ^6^ Jumbunna Institute for Indigenous Education and Research University of Technology Sydney Sydney NSW Australia; ^7^ Relationships Australia Eight Mile Plains QLD Australia; ^8^ Bouverie Centre La Trobe University Melbourne Vic. Australia; ^9^ Mercy Hospital for Women Heidelberg Vic. Australia; ^10^ Health and Social Care Unit Monash University Clayton Vic. Australia; ^11^ Molly Wardaguga Research Centre Charles Darwin University Casuarina NT Australia; ^12^ Aboriginal Studies La Trobe University Melbourne Vic. Australia; ^13^ Melbourne Poche Centre for Indigenous Health The University of Melbourne Melbourne Vic. Australia; ^14^ We Al‐li Pty Ltd Northern Rivers NSW Australia; ^15^ Social Work and Social Policy La Trobe University Melbourne Vic. Australia; ^16^ The Royal Women's Hospital, Melbourne Parkville Vic. Australia; ^17^ Women's and Children's Health Network South Australia Adelaide SA Australia; ^18^ Orygen and Centre for Youth Mental Health The University of Melbourne Melbourne Vic. Australia; ^19^ Yoo‐rrook Justice Commission Melbourne Vic Australia; ^20^ Kurbingui Youth and Family Development, Queensland Brisbane QLD Australia; ^21^ School of Psychiatry University of NSW Sydney NSW Australia; ^22^ Department of Social Work The University of Melbourne Melbourne Vic. Australia; ^23^ Murdoch Children's Research Institute Melbourne Vic. Australia; ^24^ Royal Children's Hospital Melbourne Vic. Australia; ^25^ Department of Paediatrics The University of Melbourne Melbourne Vic. Australia; ^26^ Monash Bioethics Centre Monash University Clayton Vic. Australia; ^27^ Justice and Society University of South Australia Adelaide SA Australia; ^28^ Centre for Social Research and Methods Australian National University Canberra ACT Australia; ^29^ Melbourne School of Population and Global Health University of Melbourne Melbourne Vic. Australia; ^30^ Research School of Social Sciences The Australian National University Canberra ACT Australia; ^31^ Perth Children's Hospital Nedlands WA Australia; ^32^ Division of Psychiatry University of Western Australia Crawley WA Australia

**Keywords:** Aboriginal and Torres Strait Islander, child and family services, intergenerational trauma, out‐of‐home care, prenatal notifications

## Abstract

Reducing the rate of over‐representation of Aboriginal and Torres Strait Islander children in out‐of‐home care (OOHC) is a key Closing the Gap target committed to by all Australian governments. Current strategies are failing. The “gap” is widening, with the rate of Aboriginal and Torres Strait Islander children in OOHC at 30 June 2020 being 11 times that of non‐Indigenous children. Approximately, one in five Aboriginal and Torres Strait Islander children entering OOHC each year are younger than one year. These figures represent compounding intergenerational trauma and institutional harm to Aboriginal and Torres Strait Islander families and communities. This article outlines systemic failures to address the needs of Aboriginal and Torres Strait Islander parents during pregnancy and following birth, causing cumulative harm and trauma to families, communities and cultures. Major reform to child and family notification and service systems, and significant investment to address this crisis, is urgently needed. The Family Matters Building Blocks and five elements of the Aboriginal and Torres Strait Islander Child Placement Principle (Prevention, Participation, Partnership, Placement and Connection) provide a transformative foundation to address historical, institutional, well‐being and socioeconomic drivers of current catastrophic trajectories. The time for action is now.

## 1 INTRODUCTION

Prior to colonisation, evidence suggests that Aboriginal and Torres Strait Islander children were likely to be physically, socially and emotionally healthier than European children in 1788 (Thomson, 1984). New parents were supported using principles of “Grandmothers' law” (Langton, 1997; Ramsamy, 2014). The safety and well‐being of Aboriginal and Torres Strait Islander children was fostered within systems of kinship and community care (McMahon, 2017). Since colonisation, systemic violence, removal from lands, suppression of cultural practices, racism and discrimination have caused suffering, illness and death – leaving a legacy of trauma impacting Aboriginal and Torres Strait Islander peoples (Atkinson, 2002). This systemic violence includes the forced removal of Aboriginal and Torres Strait Islander children from their families, known as the “Stolen Generations” (Wilson, 1997), which has disrupted vital kinship systems and impacted the capacity of affected parents to provide nurturing care (Chamberlain, Gee, et al., 2019). The interface between Aboriginal and Torres Strait Islander communities and governments is beleaguered by poor relationships, fragmented responsibility and conflicting priorities, all of which perpetuate inequities. The World Health Organisation's framework (Marmot et al., 2012) for understanding the causes of health inequities demonstrates how historical violence and family disruption lead to compounding cycles of intergenerational trauma (Chamberlain, Gee, et al., 2019) and childhood adversity, with major impacts on lifelong health (Anda et al., 2010; Felitti et al., 1998; Hughes et al., 2017), well‐being and prosperity (Henry et al., 2018).

In 2007, all Australian governments signed a commitment to Closing the Gap in life expectancy by improving health outcomes and equity for Aboriginal and Torres Strait Islander people with progress against key targets reported annually to Parliament. Recognition of the critical importance of early life experiences to improving health and supporting families to provide nurturing care (Britto et al., 2017) is a core element of the National Aboriginal and Torres Strait Islander health plan (Department of Health, 2013), developed to guide efforts for Closing the Gap. In July 2020, a new National Agreement on Closing the Gap was signed and, for the first time, was formed as an agreement between governments and Aboriginal and Torres Strait Islander peak bodies. This agreement included 17 national socioeconomic targets across areas that have an impact on life outcomes for Aboriginal and Torres Strait Islander peoples, including a target of reducing the rate of over‐representation of Aboriginal and Torres Strait Islander children in out‐of‐home care (OOHC) by 45 per cent by 2031 (SNAICC, 2020). Redressing compounding cycles of intergenerational trauma, a legacy of colonisation, will be central to achieving this target.

**FIGURE 1:**
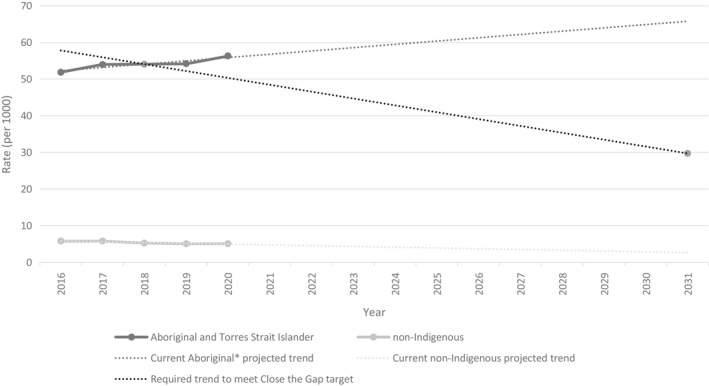
Australian children in out‐of‐home care at 30 June each year, by Indigenous status, with projections based on current trends

However, current trends demonstrate that rates of Aboriginal and Torres Strait Islander children in OOHC are continuing to increase, and were 11 times that of non‐Indigenous children on 30 June 2020 (Figure 1) (Australian Institute of Health & Welfare, 2021; Productivity Commission, 2021). While future projections based on current trends are limited, if current trajectories continue, Aboriginal and Torres Strait Islander children will be over‐represented in OOHC by more than 20 times that of non‐Indigenous children in 2031 (see illustration of projections in Figure 1). The Family Matters report estimate that, without urgent action, the number of Aboriginal and Torres Strait Islander children in OOHC is projected to double by 2029 (SNAICC, 2020).

**FIGURE 2:**
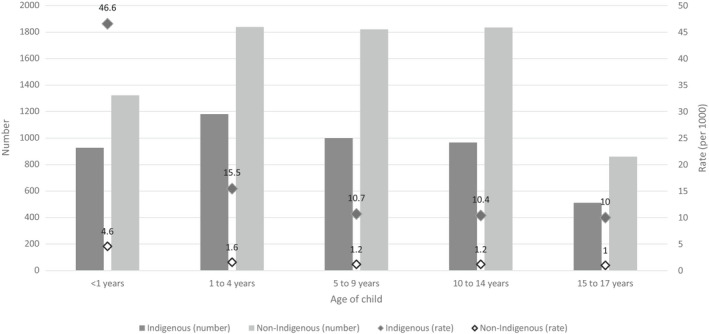
Australian children admitted into out‐of‐home care, 2019–2020, by age group and Indigenous status. *Data were extracted from AIHW Child Protection Australia data tables (2019*–*2020)* (Australian Institute of Health & Welfare, 2021)

A major contributor to these trends is the number of Aboriginal and Torres Strait Islander children being removed into OOHC shortly after birth or before one year of age, often with notifications to child protection services (CPS) before the child is born. During 2019–2020, one in five Aboriginal and Torres Strait Islander children admitted into OOHC were less than one year of age – at a rate of 46.6 per 1000 children, more than ten times the rate of non‐Indigenous infants at 4.6 per 1000 children (Australian Institute of Health & Welfare, 2021) (Figure 2).

**FIGURE 3:**
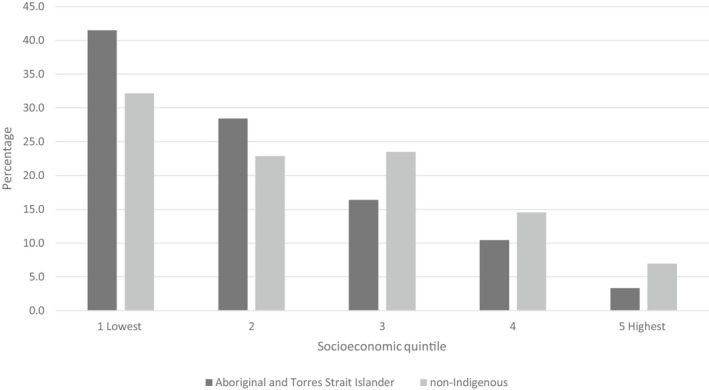
Children who were the subjects of substantiations, by socioeconomic area and Indigenous status (percentage), 2019–2020

The AIHW data highlight the importance of socioeconomic inequities, which are obscured in many other representations of these sensitive data. In 2019–2020, rates of substantiations (i.e. the conclusion, following an investigation of a notification, that there was reasonable cause to believe that a child had been, was being or was likely to be, abused, neglected or otherwise harmed) followed clear inequities across socioeconomic areas (Australian Institute of Health & Welfare, 2021) (see Figure 3). The socioeconomic gradients in substantiations were more evident among Aboriginal and Torres Strait Islander children. Socioeconomic inequities are often obscured in public health data, and it is vital that the root causes of any social or health issues are clearly illuminated, understood and addressed (Thurber et al., 2021).

Outcomes for Aboriginal and Torres Strait Islander children admitted to OOHC in Australia are very concerning. The *Our youth*, *our way*: *Inquiry into the over*‐*representation of Aboriginal children and young people in the Victorian youth justice system* report outlines a trajectory from OOHC to the youth justice system and finds significant failings of child protection and OOHC systems, in particular residential care (Commission for Children & Young People, 2021). The physical, developmental and psychological health of Aboriginal and Torres Strait Islander children in OOHC is also of significant concern (Shmerling et al., 2020). More broadly, in a longitudinal study of young Australians, 37.5 per cent reported that they had attempted suicide within four years of leaving OOHC (Cashmore & Paxman, 2007). Children removed from their parents often also experience lifelong interactions with child protection and justice systems, entrenched disadvantage and institutionalisation and disconnection from culture, community and family (Herrman et al., 2016; Tune, 2017).

The current situation can only be described as a national crisis, reflecting systemic failures, discrimination, impacts of colonisation and harmful policies (O'Donnell et al., 2019). In her *Family is Culture* report, Professor Davis questions whether Australia is meeting its obligations as a United Nations member and signatory to the United Nations Convention on the Rights of the Child (Davis, 2019), and to child welfare legislation in maintaining the best interests of the child and the integrity of Indigenous families and communities.

In this paper, we outline concerns about systemic and CPS failures regarding meeting the needs of parents experiencing social and/or emotional complexity. We argue for urgent reform and call for action for substantial investment to alter these trajectories. Our argument is bound within evidence‐based and culturally informed expertise and calls for health, social and welfare systems and their epistemological foundations to be transformed to support success rather than failure for Aboriginal and Torres Strait Islander families.

Systemic failures that do not address the needs of parents experiencing social and emotional complexity:

1. *Increasing number of Aboriginal and Torres Strait Islander children in OOHC*, *separated from families*, *communities and culture*. This does not reflect the intent of the Aboriginal and Torres Strait Islander Child Placement Principle (Davis, 2019; Guthrie et al., 2020; SNAICC, 2019, 2020) or the Australian Government Closing the Gap commitments (Australian Government, 2021).
2. *Child Protection Services (CPS) are a significant barrier for parents accessing support to prevent family disruptions and trauma for the child*. Guidelines to support pre‐birth involvement of CPS have been implemented in most Australian jurisdictions, ostensibly to enable early support for parents to prevent family disruptions and trauma for the child (Harrison et al., 2016; Taplin, 2017). Yet, fear of CPS remains a significant barrier to parents accessing support (Langton et al., 2020). CPS notifications can be triggered by a wide range of “potential risks” to the unborn child, contributing to sharp increases in infant removals in the past decade (Wise & Corrales, 2021). For example, in Western Australia, pre‐birth involvement of CPS can be triggered by a previous child being notified to the department; a family member being convicted of an offence against a child; drug and alcohol use; serious mental illness; family and domestic violence (FDV); the young age of a mother; cognitive impairment; homelessness; or the mother being in care. These blunt “screening” measures often lack specificity, which can lead to a high number of “false positives”, with significant harm caused to Aboriginal and Torres Strait Islander families. Recent analysis in New South Wales (NSW) noted that “almost 1 in 10 Aboriginal children in NSW, who entered Kindergarten in 2012, were subject to a [risk of significant harm] report before they were born” (Davis, 2019). Critically, there is no evidence whether prenatal reporting leads to improved provision of acceptable and effective support, improved outcomes for the child and mother (SNAICC, 2019), and whether it reduces the likelihood of the child being removed at or shortly after birth (Davis, 2019).
3. *Coercive practices are overused by maternity and CPS with risk of harm and triggering of protective “threat” responses*. Experts raise concerns that, in many instances, there is a risk of more harm than benefit of “interventions” for Aboriginal and Torres Strait Islander children and parents. For example, an increased focus on FDV is important, but within the context of pregnancy, this attention can be used as an additional “weapon” against mothers experiencing FDV (Langton et al., 2020). The ultimatum of requiring mothers to leave the relationship, or have her child removed, is often experienced as further systemic violence, rather than “care” supported by CPS. Perversely, threats of removal and increasing fear of losing a baby are more likely to decrease disclosure of FDV, potentially putting lives at greater risk (Langton et al., 2020). Further, there is substantial evidence to demonstrate the benefit and importance of keeping parents and their children together safely through addressing the perpetration of FDV (Cramp & Zufferey, 2020; Humphreys et al., 2020). The Power Threat Meaning Framework explains how we have learned as human beings to respond to threats that misuse power (Johnstone et al., 2018). Natural survival responses (e.g. fight, flight or freeze) are more easily reactivated or “triggered” in people who have experienced previous trauma (Blue Knot Foundation, 2021; Kozlowska et al., 2015). These “threat” or “stress” responses can negatively impact upon both maternal and foetal health (Sandman & Davis, 2012) and maternal behaviour – increasing risk and creating a harmful and dangerous reinforcing spiral. “Safety” during pregnancy and birth is critical for parents, yet antenatal health support is considered “unsafe” by many Aboriginal and Torres Strait Islander parents – because of the fear of interventions by CPS, lack of cultural safety, racism and other factors (Varcoe et al., 2013). Whatever the reason for the CPS notification, the heightened fear can also lead to reduced engagement with antenatal care, posing further risks to the unborn baby and mother during pregnancy (Sandman & Davis, 2012). Paradoxically, in some instances, a parent not attending antenatal care has been reported as grounds for CPS notification. This is despite antenatal care attendance being recognised as a woman's choice (Clinical Excellence Queensland, 2020).
4. *Systemic racism and discrimination are inherent in CPS involvement (*Edwards et al., 2021 *)*. “Risk factors” can trigger a prenatal CPS notification, and many are directly related to intergenerational trauma and the ongoing impacts of colonisation and socioeconomic deprivation (including homelessness), reflecting systemic racism. Governments and CPS fail to recognise that sustained racism and overwhelming numbers of children removed from families over generations represent cumulative harm of the system on individuals. Instead, these authorities blame the individual victims. Lack of engagement with CPS is a major “reason” for infant removal; however, the historical legacy of government policies has driven distrust of government, resulting in a lack of engagement. Yet, government services fail to take any responsibility for this situation, leaving vulnerable families to bear full responsibility with catastrophic consequences. Policy directives of statutory CPS and “regulatory ritualistic responses” (Davis, 2020) that determine practice within pregnancy and early parenting care settings mean that these factors related to systemic racism are, for too many Aboriginal and Torres Strait Islander parents, portrayed primarily as risks of harm to the unborn child (Harrison et al., 2016), rather than as social and emotional needs for support. Homelessness is a clear example of structural factors having a profound impact on families (Aboriginal Housing Victoria, 2020), which can be used as a rationale for removing children. This needs to be addressed by upstream policies.
5. *Removal of infants shortly after birth occurs without acceptable and effective support being provided to the parents*, despite CPS notification in pregnancy. Too often this occurs, without prior discussion with the parents, which is referred to within service systems as an “undisclosed infant removal” (Davis, 2019; Marsh et al., 2019; SNAICC, 2020). This unacceptable practice denies parents' information and opportunities for agency regarding their healthcare and the welfare of their infant, their right and opportunity for love, healing and recovery, and access to effective evidence‐based therapeutic support. Further, it runs counter to laws and “best practice principles” related to child protection about the need to give the widest possible protection and assistance to the parent and child, and to preserve culture. It also compounds lack of trust in the system for Aboriginal and Torres Strait Islander parents and denies their right to be involved in CPS decision‐making processes about the best interests of their infant.
6. *There is inadequate culturally safe therapeutic support available for parents with complex social and emotional needs*. The World Health Organisation defines health as a “state of complete physical, mental and social wellbeing and not merely the absence of disease or infirmity” (World Health Organisation, 1946). Healthcare services can excel in addressing a wide range of complex and emergent physical health issues (e.g. caring for preterm babies and the response to novel coronavirus). Yet, complex social and emotional health issues experienced by Aboriginal and Torres Strait Islander peoples are too often classified as “risk factors” and parents are referred “out” of health services to CPS for “support”, rather than providing culturally safe social and emotional healthcare. In a national survey of perinatal care providers, 98 per cent identified trauma, stress and grief as significantly impacting Aboriginal and Torres Strait Islander mothers, yet almost half (43 per cent) did not feel satisfied with the ability of their service to address these issues (Highet & Goddard, 2014).
7. *Vulnerable parents with complex social and emotional needs are “re*‐*victimised” by the system*, with limited or no therapeutic support provided to them after the highly traumatic and psychologically damaging experience of having their baby taken from them (Davis, 2019).
8. *CPS and maternity service documentation* of *unborn notifications* *are* *often poorly recorded and not consistent with accepted professional standards*, resulting in poor data for oversight and accountability (Davis, 2019). In some jurisdictions, such as Victoria, the Aboriginal Child Specialist Advice and Support Service (ACSASS) must be consulted at every point in CPS decision making, involving an Aboriginal and/or Torres Strait Islander child. However, mandatory requirements are not always followed, family engagement is poor, and decision‐making power still rests with CPS. Too often, critical decisions impacting the lives of vulnerable Aboriginal and Torres Strait Islander families are made without sufficient transparency, accountability, expertise, evidence and evaluation – and critically without adequately engaging parents in the process or considering the family's support needs as a priority, despite legislative and policy mandates.
9. *Healthcare providers*, *family support workers and police are exposed to a risk of “moral injury”*. Moral injury is defined as “the strong cognitive and emotional response that can occur following events that violate a person's moral or ethical code” (Williamson et al., 2021). Working within a system that harms families and does not address the complex social and emotional needs of parents places all workers at risk. Aboriginal and Torres Strait Islander workers (Davis, 2019) and young inexperienced workers (Shergill, 2018) providing direct support to mothers, babies and families are at the highest risk of “moral injury”. As well as being a serious workplace safety concern, this is likely to decrease the capacity to recruit and retain the workforce, particularly Aboriginal and Torres Strait Islander workers, many of whom are impacted by trauma in the legacies of colonisation. As Professor Davis notes in the Family is Culture Report:
  Newborn removals also pose ‘clinical, moral and ethical challenges’ for midwives, who in some circumstances question the need for the removal and resent being unable to inform the mother of an impending assumption of care. Midwives can also be frustrated at the lack of opportunity to collaborate with the [child protection] department to ensure the safety and wellbeing of the mother and her child. (Davis, 2019)
10. *Unjust treatment of and discrimination against parents who are incarcerated are widespread*. However, while access to data relating to highly vulnerable incarcerated parents is difficult to obtain (Dowell et al., 2018), the *Family is Culture* report noted that a mother's application to care for her newborn in a safe and supervised environment was denied as CPS had failed to complete a safety and risk assessment prior to the child's birth (Davis, 2019). There are limited understandings of the rights of children to be with their parents and family, and very limited opportunities to support the rights of incarcerated parents to have time with their babies. This is despite a unique opportunity to provide full‐time live‐in support, away from potentially dangerous environments and harmful influences that may have contributed to the parent being incarcerated. [Correction added on 15 February 2022 after first online publication: The word “and this” was replaced in the sentence with “that” to read as (that may have contributed.…] Evidence demonstrates that prison‐based programmes have positive outcomes for both mothers and infants (Shlonsky et al., 2016). Further evidence from longitudinal studies in the United States demonstrates that becoming a parent is the most significant lifecourse opportunity for people involved with the justice system from a young age, for recovery and escape from a trajectory of extremely poor social and emotional well‐being (Abram et al., 2017).

The interacting factors identified above have devastating and disproportionately compounding effects on Aboriginal and Torres Strait Islander families, which in turn is used to justify policy interventions (Harrison et al., 2016). For example, previous involvement with CPS places parents at higher risk of future intervention; women who are victims of family violence are “blamed” or made responsible for keeping their children safe from a violent partner or family member. Such policy positions reinforce, and are reinforced by, dominant discourses that cast difficulties that can be experienced by Aboriginal and Torres Strait Islander parents through a frame of pathologisation and/or criminalisation, supporting racist and discriminatory undertones that permeate the helping professions in community and statutory service systems (Bond, 2017). While these quotes below relate to NSW, the issues, as outlined in the *Family is Culture* report by Professor Davis (2019), strongly resonate with our authorship team.

Newborn removals are highly traumatic for the birth parents, with birth mothers recounting feelings of shock, pain, sorrow, disbelief, anxiety, guilt, shame and emptiness upon the removal of their babies. Birth mothers and fathers are left to live in an ‘in‐between state where their child is gone but did not die’, and the complexity and depth of their grief can lead to serious and longstanding psychological damage. This may then have a significantly detrimental effect on their later experiences of pregnancy and parenthood. It is widely recognised in the literature relating to compulsory child removals that many women suffer ‘a downturn in functioning’ post removal. Anecdotal evidence indicates that women may ‘seek comfort in a further pregnancy’. This may lead to successive removals of newborns from the woman's care. (Davis, 2019)

This state of affairs is unacceptable, falling far short of human rights standards and the ethical requirements of professional staff involved. Social work, maternity and early parenting support services are directly implicated in practices, which contribute to complex trauma and must take responsibility for redressing these. The current situation facing too many Aboriginal and Torres Strait Islander families is immensely harmful to babies and their families, is therapeutically unsound and unsupported by evidence, and compounds rather than resolves cycles of intergenerational trauma and disadvantage for Aboriginal and Torres Strait Islander peoples. Safe, fair, therapeutically sound and effective alternatives are available.

### 1.1 Call for action

There is unanimous agreement that the safety, love and nurturing of children are paramount (Department of Social Services, 2018). This debate centres on *the ways in which* safety, love and nurturing can and should be ensured.

Pregnancy and the first year after birth is a unique opportunity for therapeutic intervention, which is different from normal life circumstances. Evidence shows that the transition to parenting is a time of optimism and hope, offering a unique lifecourse opportunity for healing and recovery, even after severe trauma (Chamberlain, Ralph, et al., 2019). It is a known state of heightened psychological malleability thought to be partly due to hormonal changes necessary for pregnancy and attachment (Kim, 2021). Supporting parents to provide nurturing care (Britto et al., 2017) can foster a “virtuous cycle” of mutually reinforcing love, which fosters recovery from trauma through a process known as “earned security” (Segal & Dalziel, 2011). This is a key opportunity for interrupting cycles of intergenerational trauma, reducing the rate of infants admitted to OOHC, and changing current trajectories to reduce over‐representation of Aboriginal and Torres Strait Islander children in OOHC. Effective preventive interventions will also reap substantial cost savings in the high direct and indirect costs of OOHC.

The Family Matters Building Blocks (SNAICC, 2016) and five key elements of the Aboriginal and Torres Strait Islander Child Placement Principle (Prevention, Participation, Partnership, Placement and Connection) (SNAICC, 2019) provide an organising framework to coordinate a comprehensive response, with children and families at the centre. [Correction added on 15 February 2022 after first online publication: The plural form was deleted for the word “Partnerships” to read as “Partner”] We outline examples of actions under each of the child placement principles below.

1. Prevention
  Redesign maternity and neonatal services to ensure all parents have access to culturally responsive, trauma‐integrated experienced support during pregnancy, birth and early postpartum (Kildea et al., 2019). This must include community‐led, continuity‐of‐care models, which studies show can dramatically increase attendance and engagement in antenatal care – and reduce preterm births by 50 per cent (Kildea et al., 2021). Building trusting relationships between parents and professionals is the key feature of these models, which enables effective two‐way communication to enable understanding of complex social and emotional needs, and ability to access skilled therapeutic care and practical support. These culturally responsive models of care should include trauma‐integrated approaches, such as:
  - Resources to help parents understand and learn about parenting, cultural ways of fostering children's social and emotional well‐being, the effects of trauma, practical strategies to help and culturally safe support services available.
  - Access to holistic, culturally acceptable support services to foster empowerment and self‐care, offer compassionate care and support, build connections, provide parent education and opportunities to develop skills, provide practical assistance and support to develop life skills, reduce isolation and offer a range of healing and therapeutic approaches (Arabena, 2014; Austin & Arabena, 2021).
  - Education for care providers to build expertise in culturally responsive trauma‐integrated care. This includes standard training approaches to develop baseline skills and competencies, and also mentoring and supervision to build expertise and wisdom to enable best practice in supporting parents with complex social and emotional needs (Chamberlain et al., 2016). Incorporating and relearning Aboriginal and Torres Strait Islander ways of communicating effectively about sensitive issues, including using Dadirri, yarning and story‐telling, are critical (Chamberlain et al. 2020).
2. Partnership
  Aboriginal and Torres Strait Islander communities must drive the development and implementation of culturally embedded models of care for new and expectant parents, including service design and administration, Aboriginal Family Led Decision Making models (currently being evaluated in NSW and Western Australia) and other supports. Aboriginal Community Controlled Organisations should lead the design and delivery of systems, services and practice. Aboriginal and Torres Strait Islander communities have demonstrated the capacity to lead highly effective crisis responses during the COVID‐19 pandemic (Crooks et al., 2020) and are best placed to lead a comprehensive response to this complex issue (Chamberlain et al., 2016). Aboriginal and Torres Strait Islander community‐led, preventive services and solutions are highly cost‐effective (Harris‐Short & Tobin, 2019). Economic reports on the effectiveness of preventive, community‐led services demonstrate significant increased returns on investment for vulnerable Aboriginal and Torres Strait Islander families (SNAICC, 2020).
3. Placement
*Where parents are identified as needing more intensive support, all alternatives to removing the child must be explored*. This includes practical, timely and active support to address risks associated with structural inequities and socioeconomic deprivation, such as housing. This could also include enabling access to culturally safe high‐quality childcare and other necessary family supports over the perinatal and early childhood period (Sandner & Thomsen, 2018), such as the Bubup Wilam Aboriginal Child and Family Centre in Melbourne. There is a need for urgent investment in developing and evaluating pilot interventions for culturally safe, high‐quality live‐in supported parent accommodation, co‐designed with communities, which offer safe nurturing healing support. Examples such as the He Korowai Trust (2021) in Aotearoa demonstrate that this can be an acceptable strategy to provide full‐time live‐in support, and research to develop and trial these strategies is urgently needed. Using the unique opportunity to provide full‐time live‐in support for incarcerated parents to care for their infants is another important strategy to develop and evaluate a pilot programme.
4. Participation
*Parents, families and communities must be at the centre of child protection decision making*. Discussions and decision making must be transparent and open with the family in the presence of a chosen support person (e.g. Waminda's Program [NSW] and Aboriginal Family Led Decision Making pilot (Western Australian Government, 2021)), including identification of family or kin to provide care. Under no circumstances should any plans be made with hospital staff for infants to be removed from families' care without discussion and preventive plans being made with the parents and families (Marsh et al., 2019). An ethical approach demands that parents are entitled to information and opportunity for engagement in support and care and involvement in decisions about the best interests of their child. In every health service providing evidence‐based care, it is an expectation that service providers will identify risks, and strategies to mitigate these risks. The prevailing argument supporting the practice of deception around unborn CPS reports and newborn removals is that informing the parent may increase the risk that they withdraw from or try to leave maternity care (Davis, 2019). However, deeming parents to be a “flight risk” is not a justifiable rationale supported by evidence (Davis, 2019, p.189). The risk of flight and trauma is far greater where there is deception (Davis, 2019). There is no evidence that deceiving parents about plans to forcibly take their baby after birth reduces the risk of parents leaving hospital early. Rather, open, honest, transparent systems of support would allow parents and families to participate in the development of clear and attainable options.
  Consideration should be given to essential development and evaluation of a pilot model of care where CPS and perinatal care providers work together under the leadership of Aboriginal Community Controlled Organisations (The Victorian Aboriginal Children and Young People's Alliance, 2019) to develop comprehensive support plans and foster accountability, transparency and professional practice in planning support in the presence of a chosen support person (e.g. Aboriginal Family Led Decision Making). These are consistent with a therapeutic justice model of care (Marsh et al., 2019), which includes expertise and community “wise counsel” to foster access to appropriate support and ensure that complex decision‐making processes are transparent. This will help to ensure the best possible decisions are made with members of families, to increase trust in the system and build the evidence base needed for supporting parents with complex social and emotional needs. Further, close connection and intimate knowledge of the family would not be lost.
  Any notification should be accompanied by an assessment of needs and a support plan to reduce risk and provide the appropriate level of therapeutic intervention required to promote successful outcomes, whether this be for mental illness, drug use, violence, trauma or disability. The focus during this critical parenting transition should be on the current situation, rather than past concerns (The Victorian Aboriginal Children and Young People's Alliance, 2019). This is particularly salient for young parents exiting the OOHC system themselves, who frequent express fervent desire for a “fresh start” and “parenting differently” (Chamberlain, Ralph, et al., 2019). Support services must provide practical strengths‐based structural and economic support for parents to achieve these hopes and dreams of having a happy, healthy family.
5. Connection
  Where infants are removed from their parents by CPS, it is vital to ensure ongoing support for parents and families to strengthen relationships, address identified concerns and enable timely reunification. Culture remains a key feature of well‐being and resilience (Gee et al., 2014). All children have a fundamental right to connect to their family, community and culture (Harris‐Short & Tobin, 2019; Krakouer et al., 2018; United Nations, 1989). Strategies may include ensuring parents are provided with keepsakes and ways of promoting bonding with their baby (e.g. photographs, updates, clothes), support to express breastmilk if they choose to do so, and emotional support that includes fostering supportive connections. Contact arrangements need to be established as early as possible to enable connection for children to families. It is important to work holistically with the whole family to ensure that extended family and community supports are available to parents. This may include asking parents for permission to contact extended family members for support purposes, in line with culturally appropriate collective child‐rearing practices.

## 2 CONCLUSION

The current system continues to fail, and perpetuate harms inflicted on Aboriginal and Torres Strait Islander families. Culturally unsafe, inappropriate and unvalidated “risk assessment” measures; punitive approaches that prevent access to care; lack of highly skilled, trained staff to support vulnerable Aboriginal and Torres Strait Islander families; poor quality ineffective unacceptable therapeutic support options; and an absence of rigorous evaluation and evidence are driving alarming and worsening outcomes in removal of Aboriginal and Torres Strait Islander babies from their families. We must build the skills and resources to address compounding effects in this critical “window of opportunity” so that Aboriginal and Torres Strait Islander families and communities can ensure the safety and well‐being of their children.

We must commit to open and courageous truth‐telling about current child protection systems and practice, to ensure the safety and well‐being of Aboriginal and Torres Strait Islander families and children. There is a need for urgent reform to take meaningful active steps to fully implement the *intent* and recommendations of the Aboriginal and Torres Strait Islander Child Placement Principle (SNAICC, 2019), *Family Matters* Report 2020 (SNAICC, 2020) and *Family is Culture* report (Davis, 2019). We must maximise therapeutic outcomes and promote therapeutic, evidence‐based, community‐led, culturally responsive, trauma‐integrated interventions and practices. This includes identifying feasible alternatives to removing babies from Aboriginal and Torres Strait Islander parents after birth and investment in immediate implementation and evaluation.

We recognise the complexities and challenges in achieving urgently needed change in this arena of policy and practice. Dealing with complexity in perinatal care is not new. For millennia, Aboriginal and Torres Strait Islander communities have had systems for mentoring maternity care workers (traditional midwives and healers) to develop and provide technical and emotional expertise, with integrated referral systems. Perinatal services now have more trained “experts” than ever. Yet, parents experiencing social and emotional complexity are referred from the therapeutic health sector to the CPS statutory sector for “support”. We argue this creates barriers for parents accessing effective support and is punitive rather than therapeutic. If CPS involvement is required, it should be “brought in” as a participant in the multidisciplinary culturally responsive therapeutic care team to contribute to transparent shared decision‐making processes with the family.

Research is urgently needed to develop and evaluate safe, acceptable and effective responses for Aboriginal and Torres Strait Islander families that ensure the benefits of any pre‐birth support outweigh the harms and identify systemic structural issues beyond NSW. This research must be led by Aboriginal and Torres Strait Islander communities and researchers. It must also consider the rights and needs of Aboriginal and Torres Strait Islander babies, parents and families, engaging with care providers, government child protection practitioners and policymakers.

Addressing complex social and emotional needs is as challenging as addressing physical needs and is critical in the perinatal period – with strategies to improve culturally safe social and emotional care involving family support workers shown to have a significant impact on physical outcomes such as preterm births (Kildea et al., 2021). Similar investments of resources and expertise are required to achieve similar levels of success in terms of better outcomes and the associated cost savings in relation to care and support for infants and parents.

Achieving these reforms will not be easy. It requires a fundamental shift in the relationships between Aboriginal and Torres Strait Islander communities, health services and CPS, with families at the centre. Self‐determination for Aboriginal and Torres Strait Islander communities needs to be actualised – to enable communities to reassert systems of kinship and community care that foster the safety and well‐being of Aboriginal and Torres Strait Islander children. Health services need to work with communities to develop therapeutic models of care to support families with complex social and emotional health needs. Also, CPS need urgent reform – to enable Aboriginal and Torres Strait Islander communities to design and administer systems grounded in their values, perspectives and aspirations, and through them promote transparent, compassionate and healing‐focused practice with families that is consistent with social justice and reduces the incidence of moral injury. The quality of life of Aboriginal and Torres Strait Islander children caught in the CPS system depends on it, as do their prospects of a full life term. True reconciliation begins here.
